# RcAlb-PepII Perturbs the Proteomic Profile of *Cryptococcus neoformans*, Shutting Down Proteins Involved in Fungal Survival

**DOI:** 10.3390/microorganisms14081800

**Published:** 2026-08-15

**Authors:** Nicholas Silva dos Santos Filho, Lua Silva, Rossana de Aguiar Cordeiro, Patrícia Gomes Lima, Nilton Araripe dos Santos Neto, Pedro Victor da Rocha Lima, Francisco Italo Rodrigues Gomes, João Lucas Timbó Mororó, José Hélio de Araújo Filho, Felipe Pantoja Mesquita, Pedro Filho Noronha Souza

**Affiliations:** 1 Laboratory of Bioinformatics Applied to Human Health, Center for Drug Research and Development (NPDM), Federal University of Ceará, Fortaleza 60355-636, Brazil; 2 Graduate Program in Medical Microbiology, Group of Applied Medical Microbiology, Department of Pathology and Legal Medicine, Federal University of Ceará, Fortaleza 60430-160, Brazil; 3 National Institute of Science and Technology INCT Bioinspir—Bioinspired Molecules as Innovative Solutions for Agricultural Challenges, Mato Grosso do Sul 79117-900, Brazil; 4 National Institute of Science and Technology in Human Pathogenic Fungi (FunVir), Ribeirão Preto 14040-903, Brazil; 5 Postgraduate Program in Molecular Pathology, Darcy Ribeiro Campus, University of Brasília, Brasília 70910-900, Brazil; 6 Biological Science Department, State University of Rio Grande do Norte, Mossoró 59607-360, Brazil; 7 Visiting Researcher at the Cearense Foundation to Support Scientific and Technological Development, Fortaleza 60325-452, Brazil; 8 S-Inova Biotech, Graduate Program in Biotechnology, Dom Bosco Catholic University, Campo Grande 79117-900, Brazil

**Keywords:** synthetic peptides, proteomic analysis, antifungal resistance, cell death, antimicrobial peptides

## Abstract

Antimicrobial peptides (AMPs) occur naturally in living organisms and play an essential role in defense against pathogens. Consequently, these peptides are significant in public health research as alternatives in addressing microbial resistance. Here, we present the proteomic profile of *Cryptococcus neoformans* treated with RcAlb-PepII, an AMP derived from the 2S albumin of *Ricinus communis* seed cake. This research is noteworthy, as *C. neoformans* is an emerging fungal pathogen classified by the World Health Organization (WHO) as a critical threat. Proteomic analysis revealed depletion of proteins involved in DNA and RNA metabolism, reduced protein biosynthesis, and mitochondrial damage-associated proteins in *C. neoformans* following RcAlb-PepII treatment. These findings advance the understanding of the therapeutic profile of AMPs and underscore their importance in combating critical pathogens.

## 1. Introduction

*Cryptococcus neoformans* is an encapsulated fungus widely found in nature, mainly in soil and bird droppings. People commonly inhale its spores. Some animals, such as cats and farm animals, can contract this mycosis [1]. When these microorganisms enter the body, they settle in humans, colonize the lungs, and then spread throughout the body via the bloodstream [2]. Aggravating factors exist: immunosuppression, carrying HIV, and having received a transplant. Individuals for whom these factors apply have more vulnerable immune systems. Thus, when they make contact with the fungus, they are at greater risk of developing pulmonary or systemic cryptococcosis. The disease can affect the skin, kidneys, liver, and spleen in more intense forms [3].

Cryptococcal meningitis in these people remains extremely worrying, with a mortality rate reaching up to 90% [4]. Because some countries do not require compulsory reporting, the number of people affected is probably underreported [5]. Treating this fungal infection is becoming more difficult, mainly due to an increase in strains resistant to azoles, polyenes, and flucytosines. Even when used together, these drugs no longer effectively combat the disease [2,6]. This pathogen relies on several protective mechanisms: the polysaccharide capsule, melanized cells, and secretion of extracellular enzymes [2].

As a result, in 2022, the World Health Organization (WHO) published the “WHO fungal priority pathogens list” and classified *C. neoformans* as a critical pathogen due to antifungal resistance, high mortality rates, and limited therapies [7]. Along these lines, one of the most promising alternatives against resistant pathogens is the development of synthetic antimicrobial peptides (AMPs) [8]. Therefore, we used the peptide RcAlb-PepII, an α-helical, cationic peptide derived from the 2S albumin of *Ricinus communis* seed cake, which has proven efficacy against *Penicillium digitatum*, *Trichophyton mentagrophytes*, *T. rubrum*, *Klebsiella pneumoniae*, and *Candida parapsilosis*, to conduct experiments against *C. neoformans*. RcAlb-PepII showed antifungal activity against *C. neoformans*, with an MIC50 of 0.04 µg mL^−1^ and mechanisms of action that included increased membrane permeabilization, pore formation, ROS overaccumulation, followed by DNA fragmentation and apoptosis via caspase 3/9 [9]. The concentration used in this study was selected based on our previous investigations demonstrating the antifungal efficacy of RcAlb-PepII together with its favorable safety profile in mammalian cells (human fibroblasts and keratinocytes), human and rabbit erythrocytes, and zebrafish.

Therefore, this study employed proteomic analysis to examine alterations in the fungus’s protein profile. Both the presence and absence of RcAlb-PepII were evaluated, thereby enhancing the broader understanding of AMP mechanisms of action.

## 2. Materials and Methods

### 2.1. Biological Material and Chemicals

RcAlb-PepII employed in this study was synthesized by ChemPeptide (Shanghai, China) and had purity verified by high-performance liquid chromatography (HPLC) coupled to a mass spectrometer (MS). Regarding microorganisms, the yeast *C. neoformans* (ATCC 32045) was obtained from the Laboratory of Bioinformatics Applied to Human Health at the Federal University of Ceará (UFC) (Fortaleza, Brazil). All other chemicals were purchased from Sigma-Aldrich (St. Louis, MO, USA).

### 2.2. Antifungal Activity

The anticryptococcal activity of RcAlb-PepII (0.04 µg mL^−1^) against *C. neoformans* was determined in liquid yeast potato dextrose (YPD), using polystyrene flat-bottom 96-well microtiter plates, as described by the Clinical and Laboratory Standards Institute. To evaluate cell growth inhibition, 50 µL of yeast cells (2.5 × 10^6^ CFU mL^−1^) was mixed with 50 µL RcAlb-PepII (0.04 µg mL^−1^, diluted in 5% DMSO in 0.15 mM NaCl), and the minimal inhibitory concentration to inhibit 50% of cell growth was defined by [9]. After 24 h at 37 °C, cell growth inhibition was assessed by measuring absorbance at 600 nm in a microplate (Epoch, BioTek Instruments Inc., Winooski, VT, USA). The experiments were repeated three times.

### 2.3. Extraction of Proteins from C. neoformans Cells

Protein extraction from *C. neoformans* cells was carried out according to the protocol of Aguiar et al. [9]. Briefly, after the antifungal assay, the culture media were removed by washing three times with 50 mM Na^+^-acetate, pH 5.2 (extraction buffer), and the cells were centrifuged at 12,000× *g* for 15 min at 4 °C. The samples were then resuspended in 200 µL of extraction buffer and frozen at −20 °C for 24 h, vortexed for 1 min, sonicated for 30 min to break the cell wall and plasma membrane, centrifuged again, and the supernatant was collected. Protein concentration was determined using the Bradford assay [10] using bovine serum albumin (BSA). At the end, the extracted proteins were used for proteomic analysis.

### 2.4. Gel-Free Proteomic Analysis by LC/MS Mass Spectrometry Analysis

After extraction and quantification, the proteins were reduced with a 10 mM DTT solution for 1 h at 37 °C, then alkylated with 15 mM Iodoacetamide for a further 30 min in the dark. After that, proteins were incubated with Trypsin Gold (Promega, Madison, WI, USA) at a final concentration of 1:20 (*w*/*w*), as described by the manufacturer. Digestion was carried out for 16 h at 37 °C. Then, the samples were dried in a speed vacuum (Eppendorf, Hamburg, Germany) for 3 h, resuspended in 0.1% formic acid, and analyzed by nano-HPLC coupled to an ESI-QUAD-TOF mass spectrometer (Waters, Milpitas, CA, USA).

### 2.5. Protein Identification

Protein identification was carried out according to Branco et al. [11] using .pkl files from tandem mass spectra. The files were loaded into the MASCOT MS/MS ion search from MATRIX SCIENCE (https://www.matrixscience.com/cgi/search_form.pl?FORMVER=2&SEARCH=MIS, accessed on 10 August 2025). The MS/MS spectra were analyzed using the MASCOT MS/MS Ions Search platform (Matrix Science, Chicago, IL, USA). At the time of analysis, a *Cryptococcus neoformans*-specific proteome was not available among the organism-specific databases implemented in the MASCOT server used in this study. Therefore, protein identification was performed using SwissProt and the available fungal reference proteomes UP219602_*Fusarium oxysporum* and UP2311_*Saccharomyces cerevisiae*. These fungal databases were selected as reference resources for homologous protein identification, particularly because *S. cerevisiae* represents an extensively characterized fungal model with a well-annotated proteome. Protein assignments should therefore be interpreted as homology-based identifications against the reference sequences available in the databases used for the MASCOT search. During the search, parameters such as fixed modifications in Carbamidomethyl (C), variable modifications in Oxidation (O), and a peptide charge of 2+, 3+, and 4+ were set. The instrument was set to ESI-QUAD-TOF, and the following parameters were employed. The FDR was controlled at 1% and considered both fixed and variable modifications. Label-Free Quantification (LFQ) was carried out by applying minimum criteria for spectra and peptides, and the principle of maximum parsimony was used to reduce redundancy. Only proteins with at least three peptides during identification were considered, and the protein must be identified in at least two replicates out of three biological replicates to be considered.

Three groups of proteins were classified: (1) unique to the peptide-treated, identified only in the treated samples; (2) proteins unique to the control, identified only in the control samples; and (3) peptide x Control overlapping proteins. All proteins were searched and classified using the UNIPROT database.

The shared proteins between peptide x control groups were subjected to a fold change analysis with statistical significance (*p* < 0.05, Tukey’s test). Proteins with a fold-change value ≥ 1.5 (*p* < 0.05, Tukey’s test) were up-accumulated (increased abundance), and proteins with a fold-change value ≤ 0.5 (*p* < 0.05, Tukey’s test) were down-accumulated (decreased abundance) and considered for comparisons. Proteins with a fold change value between 0.5 and 1.5 were considered unchanged. The corresponding FASTA file was downloaded for each protein. The blast2go program (https://www.blast2go.com/, accessed on 30 August 2025) was used to categorize proteins by Gene Ontology (GO) annotation into molecular function, biological activity, and subcellular location.

### 2.6. Rhodamine Uptake Assay

*C. neoformans* cells were treated with RcAlb-PepII as mentioned above. After treatment, cells were washed with PBS three times, centrifuged (10,000× *g*, 4 °C, 5 min) to remove the media, and incubated with Rhodamine 123 as recommended by the manufacturer (Thermo Fisher Scientific, São Paulo, Brazil). The cells were placed on the coverslip and visualized using fluorescence microscopy on a Cytation 3 Cell (BioTek^®^, Los Angeles, CA, USA) with an excitation wavelength of 545 nm and an emission wavelength of 566 nm.

### 2.7. Statistical Analysis

All experiments were performed with three independent biological replicates. Data were analyzed by comparing group means using GraphPad Prism version 8.0 (GraphPad Software, San Diego, CA, USA). Data normality was assessed using the Kolmogorov–Smirnov test to determine whether parametric statistical analyses were appropriate. Comparisons between the control group (5% DMSO) and peptide-treated groups were performed using one-way or two-way analysis of variance (ANOVA), depending on the experimental design, followed by Tukey’s multiple-comparison post hoc test. Rhodamine 123 data were analyzed using Student’s *t*-test. Results were considered statistically significant when *p* < 0.05.

## 3. Results and Discussion

A previously published study reported that RcAlb-PepII exhibited antifungal activity against *C. neoformans*, with an MIC50 of 0.04 µg mL^−1^. The peptide exerted multiple mechanisms of action, including increased membrane permeabilization, pore formation, ROS overaccumulation, and DNA fragmentation and apoptosis via caspase 3/9 [9]. Based on that, we aimed to employ a more robust proteomic analysis to understand the effects of RcAlb-PepII on *C. neoformans* cells.

Proteomics is fundamental for understanding cell phenomena at the microscale, which are often not visible at the macro scale. By focusing on proteins, proteomic analysis can identify, quantify, and characterize interactions and functions under different conditions [12]. For these reasons, it is widely used to increase the reliability of experimental results.

**Figure 1:**
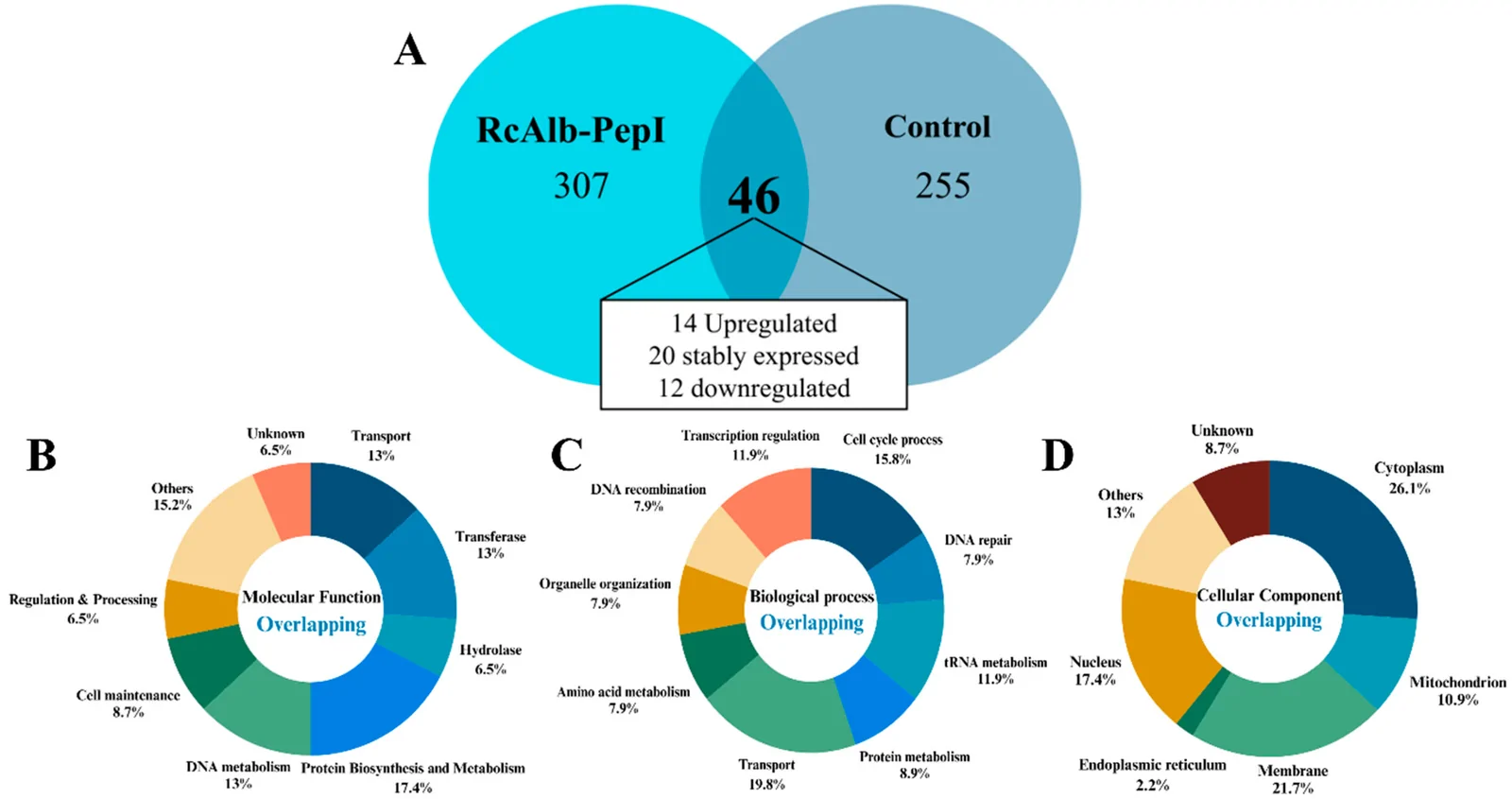
Proteomic overview of *C. neoformans* cells treated with RcAlb-PepII. (**A**) Venn diagram showing the number of proteins identified exclusively in the control group, exclusively in the RcAlb-PepII-treated group, and proteins detected in both groups (overlapping proteins). Among the overlapping proteins, 14 were up-accumulated, 20 showed no significant changes, and 12 were down-accumulated after peptide treatment. Gene Ontology (GO) analysis of the overlapping proteins showing their distribution according to (**B**) molecular function, (**C**) biological process, and (**D**) cellular component.

In this study, 301 proteins were identified in the control group, while 353 were identified in the group treated with RcAlb-PepII. Furthermore, 46 proteins were shared by both groups, and these were termed overlapping (Figure 1A and Table 1). Among the overlapping proteins, fourteen showed increased abundance, twenty showed no significant change, and twelve showed decreased abundance.

To better understand the effects of RcAlb-PepII treatment, Gene Ontology analysis was conducted to identify the molecular functions and biological processes of the proteins in this group (Figure 1B,C). Specifically, the molecular functions were divided into eight groups: Protein Biosynthesis and Metabolism (17.4%), Transferase (13%), Hydrolase (6.5%), Transport (13%), DNA metabolism (13%), Cell organization and structural maintenance (8.7%), Regulation Factor and RNA Processing (6.5%), Others (15.2%) and Unknown (6.5%) (Figure 1B,C and Table 1).

In relation to the biological processes performed by the overlapping proteins, 9 groups were identified: Cell cycle process (15.8%), DNA repair (7.9%), tRNA metabolism (11.9%), Protein metabolism (8.9%), transport (19.8%), Amino acid metabolism (7.9%), Organelle organization (7.9%), DNA recombination (7.9%), and regulation of transcription (11.9%) (Figure 1C). Regarding subcellular localization, most changed proteins are localized in the cytoplasm (26.1%), followed by the membrane (21.7%), the nucleus (17.4%), and mitochondria (10.9%) (Figure 1D).

**Figure 2:**
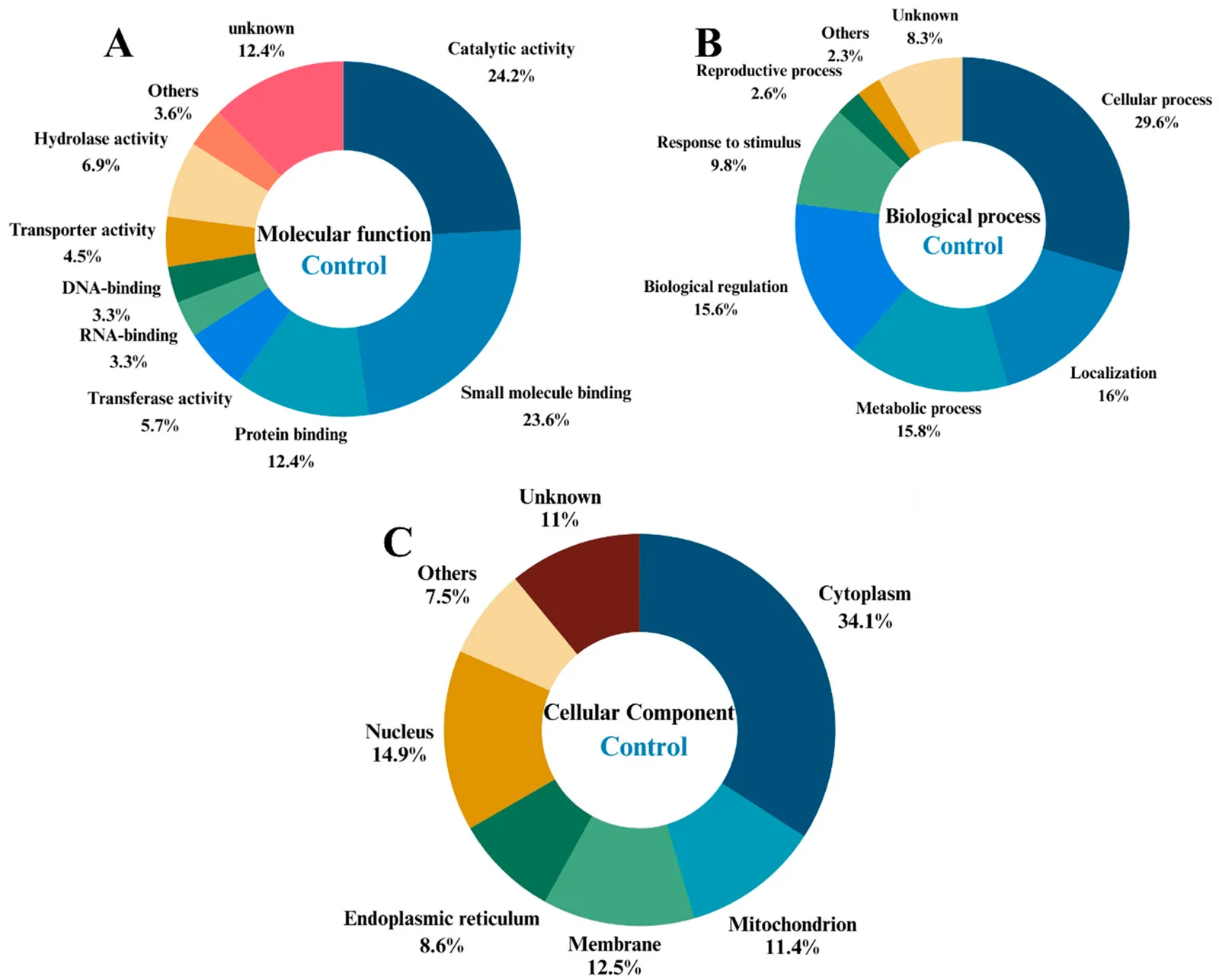
Gene Ontology (GO) classification of proteins exclusively identified in untreated *C. neoformans* cells (control group). Proteins were categorized according to (**A**) molecular function, (**B**) biological process, and (**C**) cellular component.

Molecular function analysis of proteins exclusively identified in the control group showed that they are most involved in catalytic activity (24.2%), followed by small molecule binding (23.6%) and protein binding (12.4%) (Figure 2A). Regarding biological process (Figure 2B), the majority of proteins are involved in general cellular process (29.6%) followed by metabolic process (15.8%), biological regulation (15.6%), and response to stimulus (9.8%). Regarding subcellular localization, most changed proteins are in the cytoplasm (34.1%), followed by the membrane (12.5%), the nucleus (14.9%), and mitochondria (11.4%) (Figure 2C).

**Figure 3:**
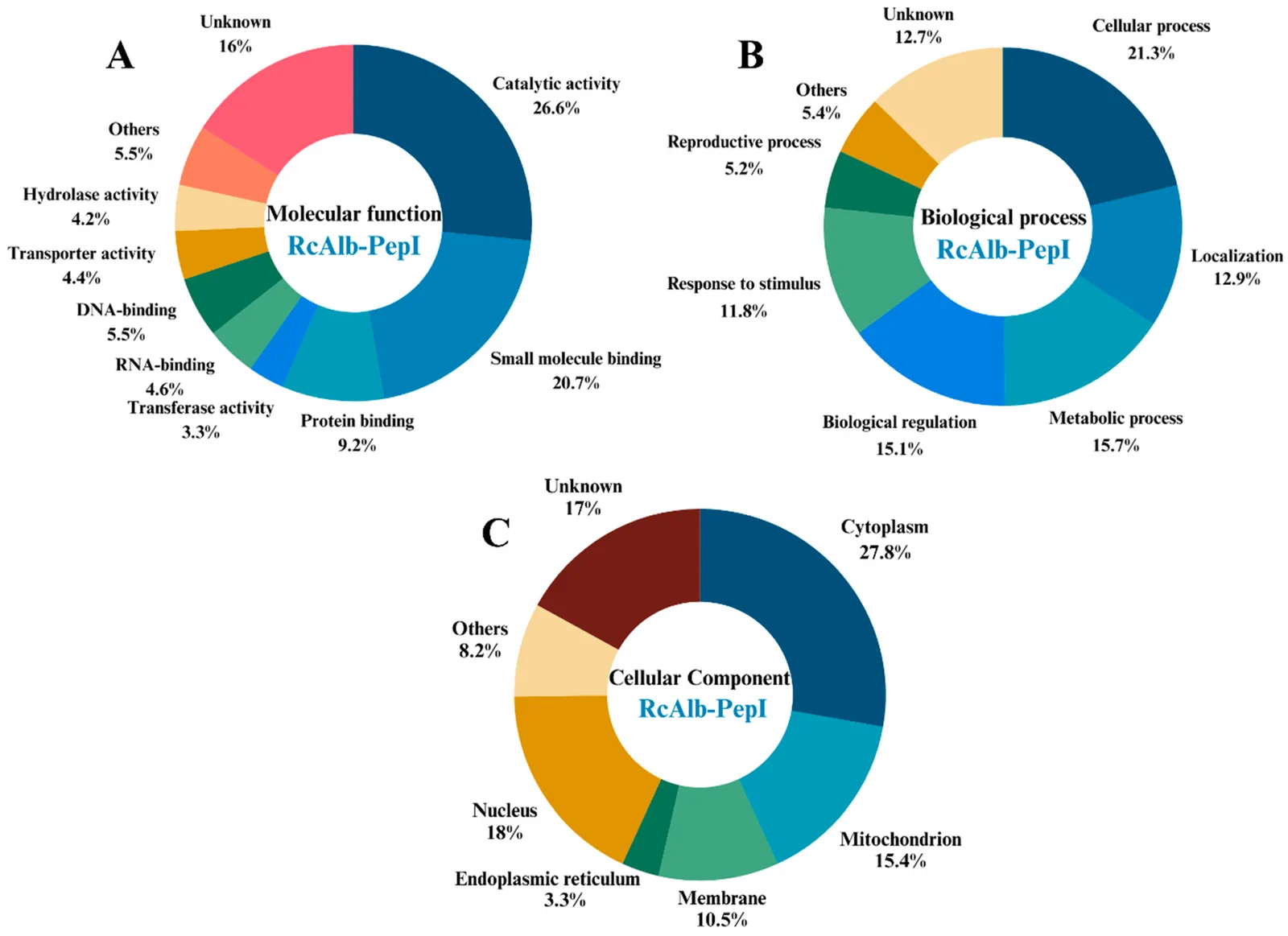
Gene Ontology (GO) classification of proteins exclusively identified in RcAlb-PepII-treated *C. neoformans* cells. Proteins were categorized according to (**A**) molecular function, (**B**) biological process, and (**C**) cellular component.

In the case of proteins exclusively identified in the *C. neoformans* cells treated with RcAlb-PepII, the molecular function revealed alterations in proteins involved in the same functions, such as catalytic activity (26.6%), small molecules (20.7%), and protein binding (9.2%) (A). Regarding biological process (Figure 3B), the majority of proteins are involved in general cellular process (21.3%) followed by metabolic process (15.7%), biological regulation (15.1%), and response to stimulus (11.8%). In the subcellular localization analysis, most changed proteins were found in the cytoplasm (27.8%), followed by the membrane (10.5%), nucleus (18%), and mitochondria (15.4%) (Figure 3C).

### 3.1. DNA Metabolism and RNA Processing Proteins

Almost all the proteins in the overlapping group classified as DNA metabolism proteins showed reduced abundance (Table 1). The exception was the sister chromatid cohesion protein PDS5, which remained stable. Notably, this group presented a 7% reduction in NB-ARC protein, which is important for nucleotide binding and ATP hydrolysis [13]. The Transcription factor domain-containing protein, responsible for regulating transcription, also decreased [14,15]. In this manner, the reduction in DNA metabolism proteins can suggests impact the cell’s growth, division, and genomic stability [16,17,18].

Moreover, the DNA damage checkpoint control protein RAD17 was present only in the cells treated with RcAlb-PepII. This protein is a component of a signal transduction system that triggers cell cycle arrest in response to DNA damage. Other proteins detected only in *C. neoformans* cells treated with the peptide included Flap endonuclease 1, serine/threonine-protein kinase CDC5/MSD2, and ATP-dependent DNA helicase PIF1, which are involved in the cellular response to DNA damage and mismatch repair [19]. The increase in the abundance of these proteins is interesting because our previous work showed that RcAlb-PepII induced ROS overaccumulation, followed by DNA fragmentation, suggesting that the cell is under stress and trying to resolve the problem caused by the peptide. Together, these findings suggest that RcAlb-PepII treatment is associated with alterations in proteins involved in DNA damage response and mitochondrial function, consistent with the cellular stress previously observed after peptide exposure.

On the other hand, mitochondrial DNA polymerase gamma (MIP1) was exclusively identified in the control cells. MIP1 is the catalytic subunit of the replicative mitochondrial DNA polymerase, and is strictly required for mitochondrial DNA replication [20]. Disruption of the MIP1 gene influences cell growth, morphology, germination, oxidative stress, and stress tolerance [21,22]. These proteomic findings are consistent with our previous observations of mitochondrial dysfunction induced after RcAlb-PepII treatment.

**Figure 4:**
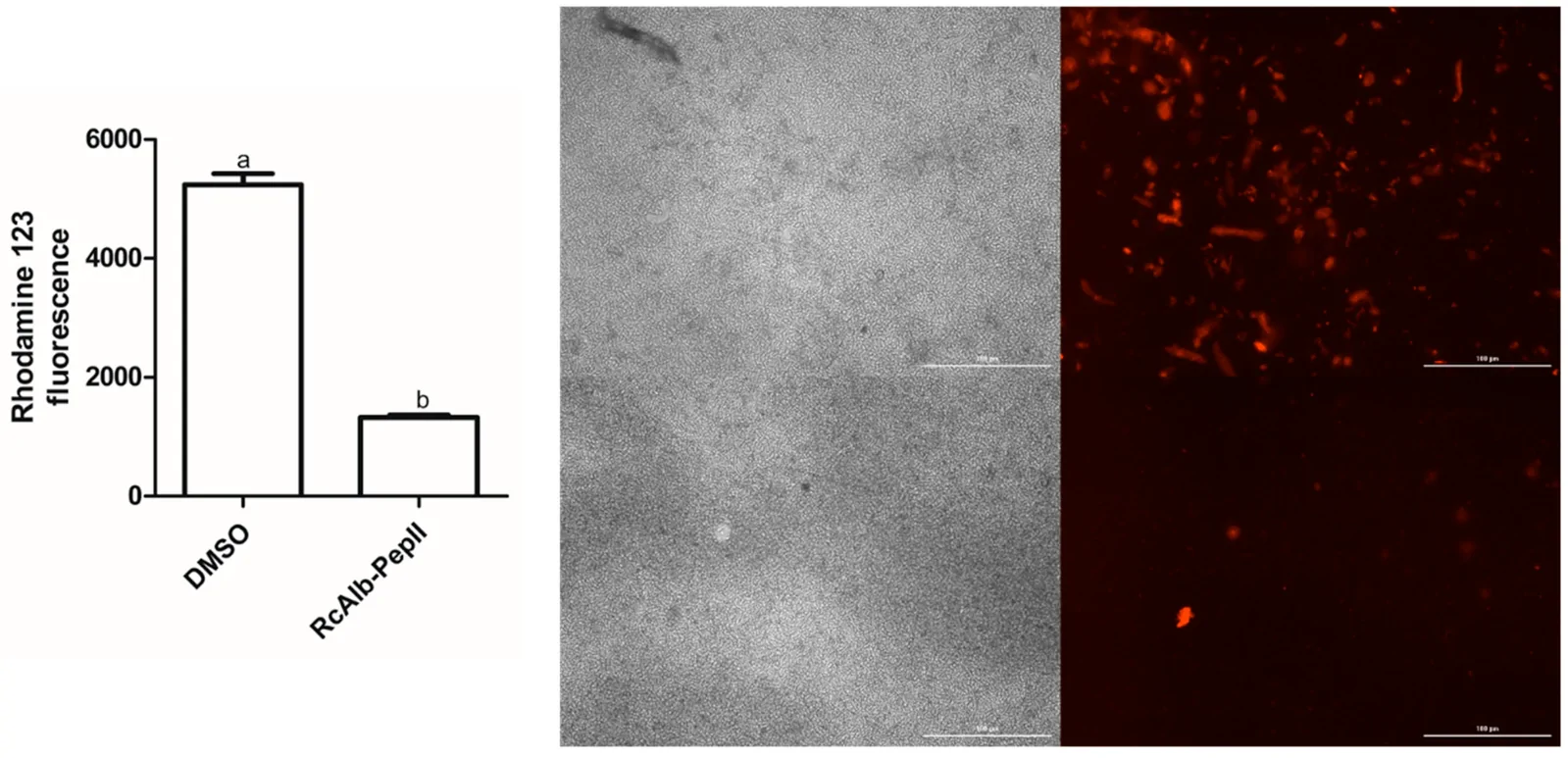
Evaluation of mitochondrial membrane potential using the Rhodamine 123 uptake assay. *C. neoformans* cells were treated with RcAlb-PepII (0.04 μg mL^−1^), stained with Rhodamine 123, and analyzed by fluorescence microscopy. Representative fluorescence images are shown alongside quantitative fluorescence intensity. Reduced Rhodamine 123 fluorescence in peptide-treated cells indicates mitochondrial membrane depolarization. Data are presented as mean ± SEM from three independent experiments. Different lowercase letters indicate statistically significant differences based on statistical analysis, which was performed using a Student’s *t*-test, with *p* < 0.05 considered statistically significant.

Additionally, to provide more evidence, an additional experiment was conducted to strengthen the idea that RcAlb-PepII causes mitochondrial damage. Therefore, the assay of Rhodamine 123 (Rhod) uptake was performed (Figure 4). Rhod is a cationic red-fluorescent dye that penetrates within healthy mitochondrial membranes. Here, it is shown that after treatment with RcAlb-PepII, Rhod fluorescence is dramatically reduced, indicating no Rhod uptake by mitochondria and thus a loss of membrane potential (Figure 4). These results are consistent with mitochondrial dysfunction previously observed after RcAlb-PepII treatment and provide additional evidence supporting the involvement of mitochondrial pathways in the fungal response to the peptide.

Other proteins identified only in the control group were the DNA cross-link repair protein PSO2, topoisomerase I, tyrosyl-DNA phosphodiesterase I, and the DNA repair protein XRS2a nuclease, all involved in DNA repair [23,24]. As reported by Aguiar et al. [9], treatment of *C. neoformans* cells with RcAlb-PepII induced DNA fragmentation, followed by apoptosis. The reduced abundance of these proteins is consistent with the DNA damage previously reported after RcAlb-PepII treatment.

Among the overlapping proteins in this group, the only decrease was in ribonuclease Z. This protein is essential for the biogenesis of functional tRNAs; thus, decreased levels of ribonuclease Z indicates a reduction in protein synthesis [25,26,27]. It is well known that ribonuclease Z can be oxidized by H_2_O_2_, leading to proteolysis [25]. Therefore, the decrease in this protein may be due to ROS-induced oxidation induced by treatment with RcAlb-PepII [9].

### 3.2. Protein Biosynthesis and Metabolism

In this group, only the Transcription factor domain-containing protein had reduced expression. This protein regulates transcription by binding specific promoters, and alterations in its abundance can affect essential features of *C. neoformans* [28]. For instance, deletion of some transcription factors in *C. neoformans* can modulate virulence factor production, antifungal drug resistance, sterol biosynthesis, and cause growth defects [28].

Conversely, the FAD-binding protein was the only protein in this group whose expression increased. The main function of the FAD-binding domain is to catalyze diverse oxidoreductase reactions, thereby playing a fundamental role in enzymatic function [28,29,30]. For instance, one important function of the FAD-binding protein Ero1p from *Saccharomyces cerevisiae* is to catalyze disulfide-linked protein folding [31]. Also, the formation of disulfide bonds in proteins can be induced by ROS, which act as signaling molecules [32]. Thus, the observed overexpression of the FAD-binding PCMH-type domain-containing protein may be related to ROS, which support the hypothesis of protein stabilization by forming disulfide bonds under stress conditions.

Previous studies showed that D-2-hydroxyglutarate-pyruvate transhydrogenase had its expression increased after treatment with RcAlb-PepII in *C. neoformans* cells [9]. However, this protein remained stable here, whereas other proteins showed varying accumulation. Elongation factor 2 (EF2) [33] is an essential protein that catalyzes ribosomal translocation during protein synthesis [34]. It is identified only in control cells. The reduced abundance of this protein may contribute to impaired protein synthesis, although functional validation will be necessary to confirm its role during RcAlb-PepII treatment [33].

The protein Rnq1 was identified only in *C. neoformans* cells treated with RcAlb-PepII. This protein aggregation may lead to prion formation [35]. Overall, yeast prions are lethal; still, some variants only slightly impair growth, as others may even be beneficial [36]. Thus, this mechanism may be related to phenotypic switching in *C. neoformans*, which possesses high genetic variability [34]. With these changes, the microorganism can adapt to different environments and also modify its virulence [34].

### 3.3. Transport-Related Proteins

The ‘anion/proton exchange transporter’ protein showed the greatest increase in relative abundance. It is related to maintaining the cell’s pH at optimal levels, especially when intracellular iron levels decrease [37]. Therefore, the transporter’s greater presence in cells treated with the peptide may reflect an imbalance in cellular pH homeostasis. Additionally, there was an increase in the accumulation of the protein GEF1, which belongs to the CLC anion transport protein family. In *C. neoformans*, these CLC proteins are involved with the production of virulence factors, capsule, and laccase [38]. Taking this into account, studies suggest that increased laccase levels in *Pichia pastoris* can enhance its resistance to oxidative stress [39].

The major facilitator superfamily (MFS) profile domain-containing protein was also overexpressed. MFS transporters are part of the largest group of secondary active transporters and export a wide range of substrates, such as carbohydrates, metabolites, neurotransmitters, nucleosides, amino acids, peptides, cations, and anions [40]. These proteins are also able to transport different drugs in various fungal species, including *Cryptococcus*, acting like drug efflux pumps [41]. Their expression can be regulated by commonly used antifungals, including fluconazole, itraconazole, ketoconazole, and caspofungin [41]. Moreover, they can also mediate resistance to oxidative stress [42]. Thus, the increase in MFS transporter suggests a mechanism of cellular resistance to SAMP and to their induction of ROS production.

The protein PHM7 participates in phosphate uptake by yeast cells. Knocking out the gene coding for this protein results in impaired phosphate surplus due to the suppression of its transport [43]. Phosphate is involved in oxidative phosphorylation and the respiratory chain, and is a cellular component of nucleic acids, ATP, cyclic AMP (cAMP), and other nucleotides [44]. Thus, a decrease in the abundance of the PHM7 protein may contribute to alterations in the essential mechanisms of the yeast cell.

Autophagy-related protein 9 (Atg9) was identified only in cells treated with RcAlb-PepII. Atg9 is a transmembrane protein responsible for the translocation of phospholipids and the formation of autophagosomes [45]. Oxidative stress induced by ROS can induce the overexpression of Atg9 genes and activate autophagy [46]. Depending on the magnitude of oxidative stress conditions caused by ROS overproduction, cellular signaling pathways may lead to apoptosis [46]. Furthermore, studies showed that Atg9 is also related to *C. neoformans* virulence [47].

### 3.4. Transferase-Related Proteins

Methyltransferase and Protein kinase domain-containing proteins were increased upon treatment with the peptide. These proteins are responsible for the methylation and phosphorylation of molecules, regulating numerous cellular processes [48]. In this manner, these proteins are involved in core cellular processes, such as DNA repair, cell cycle progression, transcription, RNA processing or translation, signaling, virulence factors, and other processes [49,50]. Specifically in *C. neoformans*, the protein kinase A participates in the coordination of phenotypic alterations of virulence factors, such as the production of capsule and melanin [51]. Thus, the overexpression of these proteins may be related to mechanisms by which fungal cells deal with exposure to RcAlb-PepII.

The acetolactate synthase regulates the catalysis of the biosynthesis of the branched-chain amino acids (isoleucine, leucine, and valine) [52]. Here, we observed a decrease in the abundance of this protein in *C. neoformans* treated with RcAlb-PepII when compared to control cells. Previous studies demonstrated that a reduction in this subunit in fungi can affect cell viability and reduce pathogenesis [53,54]. For this reason, this pathway is also a relevant drug target for antifungal development [54].

Moreover, the protein Atypical kinase COQ8 was present only in the control cells. This protein is required for the biosynthesis of coenzyme Q, involved in mitochondrial respiration, and plays a role as an antioxidant [55]. In this manner, a lack of this protein can cause respiratory deficiency [56]. Conversely, in a previous study, when treated with RcAlb-PepII, the cells of *C. neoformans* displayed an increase in abundance of this protein [9].

Cardiolipin synthase (CRD1), identified only in control cells, produces cardiolipin, a phospholipid of the mitochondrial inner membrane required for normal mitochondrial membrane potential and function [57]. Its depletion causes fragmented mitochondria, increased sensitivity to heat and antifungal drugs (caspofungin, azoles), and shows reduced respiratory growth rate [58]. On this topic, Aguiar et al. [9] reported a combined effect with itraconazole, reducing its concentration by 5× to 80% inhibition against *C. neoformans*. Overall, these observations are consistent with the increased drug susceptibility previously reported after RcAlb-PepII treatment.

### 3.5. Hydrolase-Related Proteins

Alpha-beta hydrolase (α/β-hydrolase) proteins are mainly involved in lipid metabolism. In this manner, these proteins catalyze lipid hydrolysis, thereby reducing lipid levels, such as triacylglycerol [59]. Triacylglycerols serve as reservoirs of membrane lipid precursors and also participate in the synthesis of energy and membrane lipids [60].

In this manner, the increase in α/β-hydrolase concentration may be related to lipid droplet (LD) production [61]. Stress conditions can induce an increase in LD content, performing the function of cellular detoxification, such as aiding in dealing with oxidative stress [62]. LDs might also be consumed when under stress to provide energy [63].

Found only in cells treated with the peptide, GTP-binding protein GTR1 is involved in mechanisms for protection against ROS, such as induction of autophagy to degrade oxidized organelles and macromolecules, and redox signaling for ROS excretion from the cell [64]. This is an interesting result, as shown by Aguiar et al. [9], who showed that RcAlb-PepII induced ROS overaccumulation and interfered with the activity of enzymes involved in redox metabolism.

### 3.6. Proteins Involved in Cell Organization and Structural Maintenance

The MICOS complex protein had the greatest reduction in abundance among the overlapped proteins. The proteins of the MICOS complex (Mitochondrial Contact Site and Ridge Organizing System) are found in the inner mitochondrial membrane and are responsible for joining the organelle’s ridges and organizing mitochondrial membranes [65]. Consequently, deletion of this complex affects cellular respiration, particularly the activity of respiratory complex IV (cytochrome c oxidase) [65]. Previous studies showed that cells of *C. neoformans* treated with synthetic antimicrobial peptides (SAMPs), including RcAlb-PepII, displayed an increase of Cyt c content in the cytoplasm, resulting from decoupling of Cyt c from the mitochondrial membrane [9]. As already shown, RcAlb-PepII induced a loss of membrane potential in the mitochondrial membrane (Figure 4).

MIC19 is responsible for maintaining crista junctions and the inner membrane architecture [66]. The deletion of Mic19 results in a partial loss of crista junctions, directly interfering with the electron transport chain and, consequently, cellular respiration [67]. In addition, Mic19 deficiency damages mitochondrial phospholipid metabolism, including the reduction of cardiolipin production, a phospholipid sensitive to oxidative stress and essential for the integrity of mitochondrial membrane structures [68]. Thus, it affects mitochondrial membrane organization, resulting in defects in fatty acid metabolism [68].

### 3.7. Other Proteins

Serine aminopeptidase S33 domain-containing protein was overexpressed and is predicted to perform the molecular function of triacylglycerol lipase activity [69]. Proteins in this group are associated with lysophospholipase and monoacylglycerol lipase activities [69]. Lipases are enzymes that hydrolyze triacylglycerols and are essential for lipid metabolism and pathogenicity [70]. Lysophospholipids are involved in cellular signaling processes and the regulation of cell membrane structure and can be considered a virulence factor in *C. neoformans* [71,72]. Furthermore, stress conditions, such as those induced by the SAMP treatment, may provoke *C. neoformans* cells to produce more of this protein, thereby increasing their fitness [73].

One limitation of the present study is that comparative proteomics identifies alterations in protein abundance associated with peptide exposure but does not directly identify the primary molecular target of RcAlb-PepII. Therefore, the observed proteomic changes should be interpreted as downstream cellular responses that complement previous mechanistic studies rather than definitive evidence of the peptide’s initial mode of action. Future studies integrating proteomics with target-identification approaches, genetic analyses, and in vivo models will further refine the molecular mechanism of RcAlb-PepII.

## 4. Conclusions

The present study demonstrates that RcAlb-PepII exposure is associated with extensive proteomic remodeling in *C. neoformans*. The altered abundance of proteins involved in mitochondrial function, nucleic acid metabolism, protein biosynthesis, transport, and stress responses expands the current understanding of the fungal response to RcAlb-PepII. Although comparative proteomic analysis did not establish the peptide’s primary molecular target, these findings provide complementary mechanistic evidence that is consistent with our previous biochemical and cellular studies and offer new hypotheses for future functional investigations.

## Figures and Tables

**Table 1:** Identification of overlapping proteins in the control group and the group treated with RcAlb-PepII.

Protein Identification	Uniprot Code	Reference Organism	Cellular Component	Fold Change Treated/Control Cells
DNA metabolism				
Chromosome transmission fidelity protein 18	CTF18	*Saccharomyces cerevisiae*	Chromosome	0.21
Transcription factor domain-containing protein	FOZG_17600	*Fusarium oxysporum* f. sp.	Nucleus	0.13
NB-ARC domain-containing protein	FOZG_17472	*Fusarium oxysporum* f. sp.	Nucleus	0.07
Sister chromatid cohesion protein PDS5	PDS5	*Fusarium oxysporum* f. sp.	Nucleus	1.12
Sister chromatid cohesion protein 2	SCC2	*Saccharomyces cerevisiae*	Nucleus	0.35
Zn(2)-C6 fungal-type domain-containing protein	N1SAR3	*Fusarium oxysporum* f. sp.	Nucleus	0.17
Regulation Factor and RNA Processing				
RNA cytidine acetyltransferase	KRE33	*Saccharomyces cerevisiae*	Nucleolus	2.05
Putative proline--tRNA ligase	YHR020W	*Saccharomyces cerevisiae*	Cytoplasm	1.72
ribonuclease Z	FOZG_04891	*Fusarium oxysporum* f. sp.	Mitochondrion	0.39
Protein Biosynthesis and Metabolism				
Proteasome subunit alpha type-1	SCL1	*Saccharomyces cerevisiae*	Nucleus	1.00
NADH:ubiquinone oxidoreductase intermediate-associated protein 30 domain-containing protein	FOXG_03611	*Fusarium oxysporum f.* sp.	Mitochondrion	0.55
Transcription factor domain-containing protein	FOZG_17600	*Fusarium oxysporum* f. sp.	Nucleus	0.13
Cytochrome P450	CYP505	*Fusarium oxysporum* f. sp.	unknown	1.46
FAD-binding PCMH-type domain-containing protein	FOXYS1_15356	*Fusarium oxysporum* f. sp.	unknown	4.88
D -2-hydroxyglutarate--pyruvate transhydrogenase DLD3	DLD3	*Saccharomyces cerevisiae*	Mitochondrion	1.38
C2H2-type domain-containing protein	FOZG_01868	*Fusarium oxysporum* f. sp.	Nucleus	1.13
V-type proton ATPase catalytic subunit A	VMA1	*Saccharomyces cerevisiae*	Vacule	1.35
Transferase				
Methyltransferase domain-containing protein	FOZG_11661	*Fusarium oxysporum* f. sp.	Plasm membrane	2.80
Aminotransferase	FUM8_GIBM7	*Fusarium oxysporum* f. sp.	Endosome	1.10
Protein kinase domain-containing protein	FOXB_07274	*Fusarium oxysporum* f. sp.	Cytoplasm	2.11
histidine kinase	FOXB_00653	*Fusarium oxysporum* f. sp.	Extracellular region	0.97
Fido domain-containing protein	FRV6_07135	*Fusarium oxysporum* f. sp.	Plasma membrane	1.38
Acetolactate synthase small subunit	ILV6	*Saccharomyces cerevisiae*	Mitochondrion	0.20
Transport				
Major facilitator superfamily (MFS) profile domain-containing protein	FOZG_13599	*Fusarium oxysporum* f. sp.	Plasma membrane	6.43
RNA helicase	FOZG_11103	*Fusarium oxysporum* f. sp.	Nucleus	2.00
Hypercellular protein (HypA)	BFJ69_g15177	*Fusarium oxysporum* f. sp.	Golgi apparatus	1.27
Amino acid permease/SLC12A domain-containing protein	FOZG_03819	*Fusarium oxysporum* f. sp.	Plasma membrane	1.03
Phosphate metabolism protein 7	PHM7	*Saccharomyces cerevisiae*	Plasma membrane	0.29
Anion/proton exchange transporter GEF1	GEF1	*Saccharomyces cerevisiae*	Plasma membrane	79.31
Hydrolase				
AB hydrolase-1 domain-containing protein	FOZG_11857	*Fusarium oxysporum* f. sp.	Cytoplasm	25.74
Peptide hydrolase	FOZG_15151	*Fusarium oxysporum* f. sp.	unknown	0.69
Inactive metallocarboxypeptidase ECM14	ECM14	*Fusarium oxysporum* f. sp.	Extracellular region	1.72
Cell organization and structural maintenance				
MICOS complex subunit MIC19	MIC19	*Saccharomyces cerevisiae*	Mitochondrion	0.008
Alcohol acetyltransferase FCK4	FCK4	*Fusarium oxysporum* f. sp.	Plasma membrane	0.38
Coronin	FOZG_05134	*Fusarium oxysporum* f. sp.	Cytoplasm	1.50
Bud site selection protein RAX1	RAX1	*Saccharomyces cerevisiae*	Plasma membrane	0.55
Others				
Serine aminopeptidase S33 domain-containing protein	FOYG_08533	*Fusarium oxysporum* f. sp.	Plasma membrane	7.40
Enoyl-CoA hydratase	AU210_003665	*Fusarium oxysporum* f. sp.	Cytoplasm	3.42
Secreted protein	ALP_FUSCU	*Fusarium oxysporum* f. sp.	Extracellular region	0.10
Carrier domain-containing protein	FOZG_04624	*Fusarium oxysporum* f. sp.	Membrane	0.90
Heterokaryon incompatibility domain-containing protein	FOXG_22917	*Fusarium oxysporum* f. sp.	Cytoplasm	0.50
Rhodopsin domain-containing protein	BFJ68_g9091	*Fusarium oxysporum* f. sp.	Membrane	0.64
Acyl-protein thioesterase 1	Q12354	*Saccharomyces cerevisiae*	Nucleus	0.43
Unknown				
Allergen	FOZG_14108	*Fusarium oxysporum* f. sp.	Unknown	1.30
Uncharacterized protein YMR160W	YMR160W	*Saccharomyces cerevisiae*	Unknown	0.77
HNH nuclease domain-containing protein	FOZG_17675	*Fusarium oxysporum* f. sp.	Unknown	2.54

## Data Availability

The data presented in this study are available on request from the corresponding author due to privacy or ethical restrictions.
